# Molecular dynamics simulation and docking analysis of NF-κB protein binding with sulindac acid

**DOI:** 10.6026/97320630018170

**Published:** 2022-03-31

**Authors:** Shaban Ahmad, Piyush Bhanu, Jitendra Kumar, Ravi Kant Pathak, Dharmendra Mallick, Akshay Uttarkar, Vidya Niranjan, Vachaspati Mishra

**Affiliations:** 1 International Centre for Genetic Engineering and Biotechnology, Aruna Asaf Ali Marg, New Delhi 110067, India; 2 Department of Computer Science, Jamia Milia Islamia, New Delhi 110025, India; 3 Xome Life Sciences, Bangalore Bioinnovation Centre, Helix Biotech Park, Bengaluru 560100, Karnataka, India; 4 Bangalore Bioinnovation Centre (BBC), Helix Biotech Park, Electronics City Phase 1, Bengaluru 560100, Karnataka, India; 5 School of Bioengineering and Biosciences, Lovely Professional University, Jalandhar-Delhi Grand Trunk Rd, Phagwara 144001, Punjab, India; 6 Department of Botany, Deshbandhu College, University of Delhi, Delhi 110019, India; 7 Department of Biotechnology, RV College of Engineering, RV Vidyanikethan Post, Mysuru Road, Bengaluru 560059, India; 8 Department of Biotechnology, RV College of Engineering, RV Vidyanikethan Post, Mysuru Road, Bengaluru 560059, India; 9 Department of Botany, Hindu College, University of Delhi, Delhi 110007, India

**Keywords:** NF-κB, molecular dynamics simulation, COVID-19, SARS-CoV2, sulindac sodium

## Abstract

It is of interest to document the Molecular Dynamics Simulation and docking analysis of NF-κB target with sulindac sodium in combating COVID-19 for further consideration. Sulindac is a nonsteroidal anti-inflammatory drug (NSAID) of the arylalkanoic acid class that is marketed by Merck under the brand name Clinoril. We show the binding features of sulindac sodium with NF-κB that can be useful in drug repurposing in COVID-19 therapy.

## Background:

Sulindac is a non-steroidal anti-inflammatory agent (NSAIA) that can block nuclear factor-κB (NF-κB), a transcription factor (TF) located primarily in the cytoplasm in a complex while in an inactive state. Activation of this TF makes it transition from a latent cytoplasmic form to a nuclear DNA binding state [1] and has been demonstrated to have a role in the prevention of colon cancer [2] by its inhibition [1-3].This TF is known to selectively inhibit interferon-gamma-induced expression of the chemokine CXCL9 gene in mouse macrophages [4]. The growth inhibitory and anti-inflammatory properties of sulindac are possibly due to its ability to reduce prostaglandin synthesis by cyclooxygenase inhibition [2]. Furthermore, Yamamoto et al. [2] have demonstrated that aspirin and sodium salicylate inhibited the activity of an IκB kinase β (IKKβ) that is required to activate the NF-κB pathway, which leads to nuclear translocation of NF-κB, where it binds to its cognate DNA and activates transcription of a wide variety of genes involved in host immunity, inflammation, cell proliferation and apoptosis [2,3]. Various NF-κB inducers are known to be highly variable and include aggregates of bacterial lipopolysaccharides, ionizing radiation, reactive oxygen species (ROS), cytokines, such as tumour necrosis factor alpha (TNF-α) and interleukin 1-beta (IL-1β) and viral DNA and RNA [4]. Upon activation, NF-κB promotes the gene expression of a broad range of cytokines (e.g., IL-1, IL-2, IL-6, IL-12, TNF-α, LT-α, LT-β, and GM-CSF), chemokines (e.g., IL-8, MIP-1, MCP1, RANTES, and eotaxin), adhesion molecules (e.g., ICAM, VCAM, and E-selectin), acute phase proteins (e.g., serum amyloid A: SAA), and inducible effector enzymes (e.g., inducible nitric oxide synthase: iNOS and cyclooxygenase-2: COX-2). The TF NF-κB can be considered as a "quick action" primary transcription factor that is able to regulate myriad of cellular responses, including the host's early innate immune response to infection, and is also associated with chronic inflammatory states, viral infections, septic shock syndrome and multiorgan failure [5,6] viz-a-viz ascribed as an anti-apoptotic TF [7].The constitutive activation of NF-κB pathways has been responsible for stimulation of inflammatory diseases, such as multiple sclerosis and rheumatoid arthritis [8,9]. Furthermore, p38 MAPK-based activation of NF-κB has also been reported [9]. Drug induced suppression of NF-κB has been found to have a strong potential in proapoptotic signal modulation therapy [10].

Recently, a study utilizing molecular dynamics simulations (MDS) - based analysis targeting human angiotensin-converting enzyme 2 (hACE2) as a receptor showed interesting results on development of a lead candidate to be used later as a therapeutic drug against COVID-19 [11] Though in the current scenario, the spread of COVID 19-based pandemic has slowed down to a great extent, but its resurgence cannot be ruled out. Due to the lack of adequate specific treatments for COVID-19, there is an urgency to develop or repurpose drugs to help end the epidemic completely. The drug discovery process has received a great impetus with the emergence of computer-aided drug discovery (CADD) and has been instrumental over the last decade in exploring protein inhibitors in protein-drug interactions and protein–protein interactions [12]. Since the process involved in the development of a candidate drug into an approved drug is lengthy and expensive, a combination of computational methodologies such as virtual screening, docking, molecular dynamics simulation, and binding free energy evaluation contributes to identifying potential drug candidates from compound libraries [13]. To date, MDS and structure-based virtual screening studies have been carried out to understand the SARS-CoV-2 spike protein functions [14-16], role of repurposed protease inhibitors in blocking SARS-CoV-2 [17], effects of some antiviral drugs on SARS-CoV-2 [18], effect of temperature on the structure of the spike protein [19], SARS-CoV-2-targetted potent drugs that can be effective in controlling COVID-19 [20-21] as well as many other aspects of biology qualifying to control SARS-CoV-2 progression. However, the rate of occurrence of different pathological human phenotypes and their heterogeneous lethality rates arising from constant mutations of SARS-CoV-2 [22-23] indicate serious problems that need to be appropriately dealt with. A paradigm shift in the strategy on targeting COVID-19 borne biomolecules-for therapeutic purposes to human-based molecular biomarkers is much necessitated in the current scenario, since the current approaches based on former fail to yield appreciable outcomes. A recent study revealed the importance of the NF-κB pathway in developing therapy regimens for critical COVID-19 patients [24]. Other reports have also substantiated the above fact by arguing that therapeutic benefits of NF-κB inhibitors, including dexamethasone, a synthetic form of glucocorticoid, have increasingly been realized now [17]. Abnormal activation of NF-κB resulting from SARS-CoV-2 infection might be associated with the pathogenic profile of immune cells, cytokine storms and multiorgan defects [23]. Therefore, we currently explored the structural details of NF-κB via an in silico approach wherein we pinpointed the protein pockets for the binding of SARS-CoV-2 spike proteins. A hypothetical explanation currently generated is that upon entering the human body, the COVID-19 viral protein might be triggering the action of the NF-κB directly or through activation of IKK, the kinase phosphorylating IκBs that otherwise remain bound with NF-κB to keep it in an inactive state in the cytoplasm. This argument is made in the light of the fact that another complex member, IκBα is known to be phosphorylated by specific kinases at two sites near the N-terminus (Ser-32 and Ser-36), while the phosphorylated protein is then ubiquitinated at Lys-21 and Lys-22, leading to proteosome-mediated degradation [24] and release of NF-κB from the complex. The removal of IκB unmasks the nuclear localization sequence (NLS) of NF-κB and allows its translocation to the nucleus [24]. Thus, IκBα is a multifunctional inhibitor of NF-κB that blocks nuclear translocation, DNA binding, and phosphorylation by protein kinase A (PKA). Inhibition of NF-kB blocks the NF-κB-mediated deactivation of immune and inflammatory responses to stimuli, such as cytokines or bacterial/viral infection products [24]. Along with NF-κB, sulindac has been of interest because of its many roles that includes also as a chemo preventive agent for adenomatous colorectal polyps and colon cancer [25] We show here that sulindac is able to bind specifically to a pocket on NF-κB, a concept that becomes theoretically important for exploring its therapeutic potential [26-29].

## Materials and Methods:

### Protein and Ligand Preparation:

Coordinates of the X-ray diffraction-based crystal structure of the I-κ-B-α/NF-κB complex with solvents (PDB ID: 1NFI) at a resolution of 2.70 Å were downloaded from the Protein Data Bank (PDB). Solvent molecules and chains A, B, and F were removed during protein preparation using the Protein Preparation wizard from Schrodinger Maestro (Schrodinger, LLC, and New York, NY, USA). The remaining structures were processed using the Protein Preparation wizard using appropriate methodologies. The SARS-CoV-2 structure possessing a loop (residues 376-381) was missing in the PDB structure and hence was modelled using Schrödinger's Prime module. Hydrogen atoms were incorporated, and a standard protonation state at pH 7 was used. Bond orders were assigned using the chemical components dictionary (CCD) database. The heterostate was generated using Epik with a pH of 7 (±2.0), and Prime was used to fill the missing chains and loops. While refining the structure, PROPKA with pH 7.0 was used along with the sample water orientation. While minimizing the structure, a root mean square deviation (RMSD) of 0.30 Å with the OPLS_2005 force field was used. The ligand was selected based on published information regarding its use as a therapeutic molecule for various human diseases. The ligand was prepared using the Ligprep wizard of Schrodinger with the OPLS_2005 force field; the Epik job was submitted with the metal-binding site and included the original state. Thirty-two stereoisomers were assigned per ligand with specified chirality's. Glide was used to filter the search to locate the ligand in the active-site region of the receptor. The shape and properties of the receptor were represented on a grid to provide a more accurate scoring of the ligand poses. The docked complexes were superimposed on the original crystal structure to calculate the RMSD (Figure 1 - see PDF).

### Virtual Screening and Molecular Docking:

The Glide grid generator was used for generating the grid for blind docking. A Schrodinger virtual screening workflow (VSW) was used to score the virtual screening with default parameters employing the Glide program of Schrödinger. Use of HTVS mode allowed the elimination of most of the stereoisomers, and only 10% of the total ligands could be retained that passed the screening. Ligands following QikProp and Lipinski's rule were filtered for docking. Docking in the VSW was performed with Epik state penalties and further passed through HTVS, SP and XP docking modes. The interactions of the selected ligand and protein docked complexes were analyzed by a pose viewer.

### Molecular Dynamics Simulation:

To study the dynamic behavior of the protein complex under simulated physiological conditions, MDSs of the protein-ligand (PL) complex were performed using Desmond, which is available with Schrodinger Maestro (v12.5). The PL complex (9873 atoms) was solvated in a 10 x 10 x 10 Å orthorhombic periodic box built with TIP3P water molecules [30]. The whole system was neutralized by adding an appropriate number of 6 Na+ counterions. This solvated system was energy minimized and position restrained with OPLS_2005 as the force field. Furthermore, 100 ns of MDS was carried out at 1 atm pressure and 300 K temperature, implementing the NPT ensemble with a recording interval of 100 ps, resulting in a total of 1000 read frames. Finally, various parameters of the MDS, such as ligand-binding site analysis, RMSD, root mean square fluctuation (RMSF), PL contacts, secondary structure element (SSE) analysis, etc., were also analyzed to check the stability, compactness, structural fluctuations and PL interactions in the solvated system (Figure 2 - see PDF).

### Interaction Analyses:

#### Structural Data:

The PDB was used to extract all 3-D structural information. The X-ray diffraction-based crystal structure of the I-κ-B-α/NF-κB complex with solvents (PDB ID: 1NFI) at a resolution of 2.70 Å was used for this study. Solvents and chains A, B, and F were removed during protein preparation. The structure of the SARS-CoV-2 spike glycoprotein (closed state) solved using electron microscopy (PDB ID: 6VXX) with a resolution of 2.80 Å was obtained. Furthermore, all water molecules were removed from both structural data sets. The educational version of PyMOL (The PyMOL Molecular Graphics System, v1.2r3pre, Schrödinger, LLC) was used to generate the images derived to understand and analyse the structure and inter chain interaction information.

#### Molecular Docking:

The ligand sulindac chosen in the current study for the mentioned reasons has its docking score and binding affinity shown in Figure 3(see PDF). The complex of the drug molecule docked in the pocket of NF-κB was designated S0 (Figure 4A - see PDF) and was taken as the reference for further interaction analysis.

#### Pocket Analysis:

The molecule NF-κB was found to having multiple pockets, as calculated using the CASTp 3.0 server [30-31] Three largest pockets (based on the accessible area) of these were considered for analysis. The residues present in these pockets interacted with the spike protein on exposure changing the area of the pockets significantly due to this interaction and also upon the drug interaction or the combination of the spike protein and the drug together.

#### Protein-Protein Docking:

Protein-protein docking studies were carried out using ClusPro 2.0 [32] with minor changes following the protocols used in Jorgensen et al. [33]. This job was carried out to understand the interaction of the SARS-CoV-2 spike glycoprotein with NF-κB in the presence and absence of the drug molecule. The best pose of the spike protein binding with the NF-κB-IkB complex was selected based on the cluster size, which is how the models are ranked in ClusPro. S1 (Figure 4B - see PDF) represents the complex of NF-κB with the SARS-CoV-2 spike glycoprotein, and S2 (Figure 4C - see PDF) represents the complex of S0 with the SARS-CoV-2 spike glycoprotein.

LigPlot+ v.2.2 [34] was used to find the ligand-protein interaction of complex S0 (Figure 2 - see PDF). PDBsum was used to calculate the interaction between NF-κB and IkB (Figure 5A, 5B - see PDF), the interaction between the spike protein and NF-κB (Figure 5B - see PDF) and the interaction between the spike protein and NF-κB docked with drug molecules. PyMOL was used to analyse the structural differences imposed on NF-κB when it interacted with the drug molecule and the spike protein in the presence of the drug molecule.

#### MM-GBSA Studies:

Prime MM-GBSA (molecular mechanics, the generalized born model and solvent accessibility method) calculations were performed with the docked complex of receptor and sulindac. The implicit solvent model used is VSGB [35] which has an efficient approximation of the solvation free energy with Surface Generalized Born (SGB) over Vacuum and chloroform solvation models. The following calculations were made

1 Rec Strain = Receptor (from optimized complex) - Receptor

2 Lig Strain = Ligand (from optimized complex) - Ligand

3 MMGBSA dG Bind = Complex - Receptor - Ligand

4 MMGBSA dG Bind (NS) = Complex - Receptor (from optimized complex) - Ligand (from optimized complex) = MMGBSA dG Bind

#### Rec Strain - Lig Strain:

NS here refers to binding or interaction energy without any involvement of conformational changes occurring in the receptor or ligand. The potential energy of the complex will be reported in kcal/mol.

## Results and Discussion:

### MD Simulation Studies:

The complex RMSD in the initial phase at 1.63 Å went to 4.11 Å while stabilizing the structure for 100 ns of simulation. Initially, at up to 50 ns, the RMSD did not fluctuate much, but after that, the stability fluctuated for 10 ns and stabilized subsequently (Figure 1 - see PDF). The RMSF plot analysis displayed minimal fluctuations in the protein structures (Figure 2 - see PDF). Furthermore, minimal fluctuations were also observed in the PL complex, and the RMSF plot showed fluctuations in some regions of the protein. The results of the residue interaction analysis after docking are highlighted with specific colours based on the self-explanatory features (Figure 3 - see PDF). The docking interactions of sulindac (Figure 4A, 4B - see PDF) showed that it docked in the largest pocket of the NF-κB-IκB complex that contains most of the residues interacting with the spike protein. It is possible that drug binding to the complex interferes with the above interaction of the spike protein, however, needs to be ascertained with more robust wet laboratory-based experimental designs. The sulindac was found to bind to other pocket than the one occupied by NF-κB-IkB complex (Figure 4C - see PDF). Moreover, data shows the importance of NF-κB as a suitable drug receptor for COVID-19 for its proven roles as molecular targets in drug discovery-based in silico studies (Table 1 - see PDF).

The interaction made by the drug molecule with the residues of the active site cavity of NF-κB, IkB and P50 peptide chains is dynamic in nature and formed, broke and reformed during the simulation duration (Figure 3 - see PDF). A brief duration of 20 ns was observed when no interaction occurred between the drug molecule and the NF-κB active site residues. Although there was an interaction between the ligand (drug) molecule and residues of NF-κB (chain C) and IκB (chain E) in the first 50 ns, the interaction between the drug and IκB (chain E) disappeared after 50 ns. This loss of interaction strengthened the ligand-NFkB interaction.

The changes in the 3-D structure of the NF-κB-IκB complex due to the binding of the drug molecule and spike protein in the presence of the drug molecule were compared to that of the unbound complex (Figure 5A - see PDF). The binding of the drug molecule to the NF-κB-IkB complex led to an increase in the compactness of the complex. The area and volume data of the pockets presented in Figure 5A(see PDF) corroborated this observation. The binding of the spike protein in the presence of the drug molecule further increased the compactness of this complex, possibly due to increased interaction between the chains of the NF-κB-IκB complex. Additionally, we made a comparison of the amino acid (aa) residues of the spike protein, MAP kinase and pLC gamma during their interactions with the residues of the NF-κB molecule to identify if any common pattern existing in aa selection by sulindac during these interactions. However, all three sets of interactions, such as NF-κB-sulindac docked with spike protein, NF-κB sulindac docked with MAPK and NF-κB sulindac docked with pLC gamma had different aa residues interacting with the chosen ligand (sulindac)(Figure 6 - see PDF), revealing all these as independent events. In our studies, MM-GBSA studies on docked complexes revealed dG bind values that were reasonably in agreement with ranking based experimental scores, i.e., binding values We first calculated the MMGBSA dG Bind = Prime Energy (Optimized Complex) - Prime Energy (Optimized Free Ligand) - Prime Energy (Optimized Free Receptor) and second, MMGBSA dG Bind(NS) = Prime Energy (Optimized Complex) - Prime Energy (Ligand Geometry From Optimized Complex) - Prime Energy (Receptor Geometry From Optimized Complex). The values were found to be -2.15 kcal/mol for MMGBSA dG bind and -2.71 kcal/mol for MMGBSA dG bind (NS). The atoms contributing to the binding affinities are shown in Figure 7(see PDF).

Our understanding on the involvement of NF-κB signalling pathway in COVID-19 is limited, but NF-κB inhibitors have been increasingly utilized to gain therapeutic benefits in many human diseases [49-50]. Abnormal activation of NF-κB in the manifestation of SARS-CoV-2 infection is reported for the pathogenic profile of immune cells, cytokine storms and multi organ defects and cytokine storm alone is capable of triggering excessive inflammatory response to SARS-CoV-2 and is extremely responsible for disease severity [51]. Thus, pharmacological inactivation of the NF-κB, such as proposed currently in the use of sulindac, NF-κB-kinase interaction either directly or by inhibiting the dephosphorylation of its inhibitors, such as IKKα, IKKβ and other associated molecules, strongly represents a potential therapeutic target to treat the symptoms of COVID-19 in clinical trials. A survey of literature since the emergence of COVID-19 clearly reveals that majority of the drugs formulated to tackle SARS-CoV-2 thus far, have been structured to target virus [52]. However, given the current situation where the virus mutates so rapidly, our conceptual paradigm on targeting the key viral proteins through drug intervention need to get changed towards the host proteins, such as human proteins that hold key portfolio in SARS-CoV-2 - mediated infections. This would possibly take care of the various mutants of SARS-CoV-2 that have been difficult to control until now. Furthermore, cytokine storm in COVID-19 [53] and various drugs that were initially used for therapy need to be revisited in light of modern researches based on drug repurposing, which are potentially very effective in treatments of even the anomalies arising post-COVID-19 infection and can thus counteract the many aberrant responses thereof.
